# Electronic structures of defects and magnetic impurities in MoS_2_ monolayers

**DOI:** 10.1186/1556-276X-9-676

**Published:** 2014-12-13

**Authors:** Shang-Chun Lu, Jean-Pierre Leburton

**Affiliations:** Department of Electrical and Computer Engineering, and Beckman Institute, University of Illinois at Urbana-Champaign, Urbana, Illinois 61801 USA

**Keywords:** DFT, MoS_2_, P-type dopants, Magnetic impurities, Density-of-states, Formation energy, **PACS:**, 73.20. -r, 73.20.At, 73.20.Hb

## Abstract

We provide a systematic and theoretical study of the electronic properties of a large number of impurities, vacancies, and adatoms in monolayer MoS_2_, including groups III and IV dopants, as well as magnetic transition metal atoms such as Mn, Fe, Co, V, Nb, and Ta. By using density functional theory over a 5 × 5 atomic cell, we identify the most promising element candidates for p-doping of MoS_2_. Specifically, we found VB group impurity elements, such as Ta, substituting Mo to achieve negative formation energy values with impurity states all sitting at less than 0.1 eV from the valence band maximum (VBM), making them the optimal p-type dopant candidates. Moreover, our 5 × 5 cell model shows that B, a group III element, can induce impurity states very close to the VBM with a low formation energy around 0.2 eV, which has not been reported previously. Among the magnetic impurities such as Mn, Fe, and Co with 1, 2, and 3 magnetic moments/atom, respectively, Mn has the lowest formation energy, the most localized spin distribution, and the nearest impurity level to the conduction band among those elements. Additionally, impurity levels and Fermi level for the above three elements are closer to the conduction band than the previous work (PCCP 16:8990-8996, 2014) which shows the possibility of n-type doping by Mn, thanks to our 5 × 5 cell model.

## Background

Recently, two-dimensional (2D) materials have attracted intensive attentions due not only to the rich and fundamental physics brought by them but also to their potential for nanoscale device applications [1]. Graphene [2–5] is the most well-known member in the family of 2D materials, but its gapless band structure has been deemed as a considerable drawback for realizing switching operation, which is essential for digital logic devices. Even though the bandgap of graphene can be engineered by depositing on particular substrates [6] or fabricating nanoribbons [7, 8], it deteriorates the mobility. For this reason, researchers have turned to other kinds of 2D materials called transition metal dichalcogenides (TMDCs) [9]. These materials can also be exfoliated into 2D layers from their stacked crystal structure by using the same method as for graphene production [10]. Most intriguingly, their band structures are layer-thickness-dependent despite the weak interlayer van de Waals forces, which indicates they are electronically tunable via thickness control. As the number of layer reduces from bulk value to monolayer, the bandgap of several TMDC materials changes from indirect to direct [11]. Among those and the most widely studied materials, monolayer MoS_2_ has emerged as a semiconducting alternative to graphene because of its large intrinsic direct bandgap of approximately 1.8 eV [12], which makes it suitable for optoelectronic and nanoelectronic applications. In addition, easy fabrication using exfoliation method, absence of interface dangling bonds, and superb electrostatic behavior are the main reasons why MoS_2_ is the subject of nanotechnology research and is a competitive candidate for logic devices of the next generation [10]. Recent experimental works have shown transistors made of single-layer or few-layer unintentionally doped MoS_2_ exhibiting very high on/off ratios, exceeding 1 × 10^8^, close-to-ideal SS (approximately 70 mV/dec), ultralow standby power, and mobility of at least 100 cm^2^/Vs [10] or even up to 700 cm^2^/Vs when high-k dielectrics are applied [13], which is competitively comparable to those of current Si-based CMOS technology. On the other hand, good performances were also observed in unintentionally doped multi-layer MoS_2_ transistors [14].

Many unintentionally doped MoS_2_ transistors reported in the literature show either n-type [10] or p-type [15] behaviors, with their related defects or impurities altering the transport properties. Although simulations of the electronic and transport properties of MoS_2_ containing various dopants [16, 17] have been reported, to the best of our knowledge, there is still lack of comprehensive and coherent understanding of the groups III and IV dopants on MoS_2_, even in the most recent reports [16]. For magnetic impurities, although there are a number of theoretical reports on Mn, Fe, and Co [17, 18], our model on a 5 × 5 computational cell shows impurity levels and the Fermi levels located closer to the conduction band by about 0.1 ~ 0.2 eV and ~0.2 eV, respectively. In this paper, we provide a systematic study on the properties of various p-type dopants, vacancies, and magnetic impurities in monolayer MoS_2_ including group III dopants, i.e., B, Al, and Ga and group IV dopants, i.e., C, Si, and Ge, as well as magnetic transition metal elements such as V, Nb, and Ta. For the first time, the electronic properties of groups III and IV elements are studied comprehensively in a 5 × 5 simulation supercell of single-layer MoS_2_ in addition to the investigation of a large number of magnetic transition metal elements on their electronic states, formation energies, and spin properties. Moreover, Mo as an adsorbate in MoS_2_ is considered. Our objective is to identify the most promising candidates of p-type dopants and magnetic impurities for MoS_2_. The information will come in handy when fabricating doped devices such as field-effect transistors (FETs), optoelectronic devices, and all those requiring p-n junctions. In addition, the study of spin distribution around magnetic elements provides the basic knowledge for future applications of MoS_2_ spintronics, which is one of the possible scenarios in the beyond-CMOS technology.

## Methods

In this work, density functional theory (DFT) is employed to perform *ab initio* calculations on the electronic and magnetic properties of monolayer MoS_2_ doped with impurities using the Quantum ESPRESSO software package [19].

**Figure 1:**
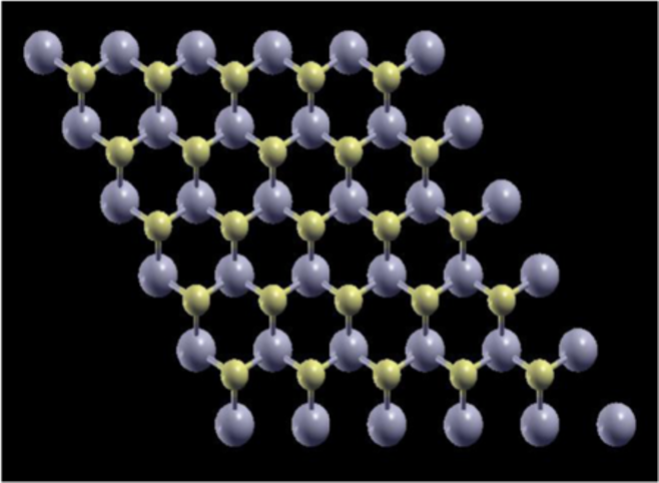
**Crystal structure of monolayer MoS**_**2**_ **(blue: Mo, yellow: S).**

Ultrasoft pseudopotentials are chosen for our simulations, which are carried out for a 5 × 5 × 1 hexagonal supercell (lateral dimension fixed to 15.83 × 15.83 Å^2^) with 25 Mo atoms and 50 S atoms (Figure 1). At the beginning, 3 × 3 × 1, 5 × 5 × 1, and 7 × 7 × 1 supercells are all taken into consideration. However, after some calculations, we find the calculated impurity states and formation energies stabilize at 5 × 5 × 1 cell size; therefore, it is chosen for the balance between calculation accuracy and computational costs. A 12-Å vacuum layer is introduced in our slab model to prevent the interaction between neighboring supercells and mimicking the realistic condition of monolayer MoS_2_, and periodic boundary conditions are adopted. Doping (defects) in MoS_2_ is introduced by replacing (removing) a single host atom in the system. The resulting doping or defect concentration is 4% (2%) as Mo (S) atom is replaced or removed. A high cut-off energy of 816 eV for wave functions and a dense 5 × 5 × 1 Monkhorst-Pack k-point mesh over Brillouin zone are used for the geometric optimization with the generalized gradient approximations (GGAs) in the Perdew-Burke-Ernzerhof parameterization [20]. The relaxation is going on until all components of all forces in the system are smaller than 1.03 × 10^−2^ eV/Å and the total energy of the supercell converges to less than 1.36 × 10^−5^ eV. With spin information acquired in the geometric optimization, spin-polarized density of states (DOS) calculations are continued on the structures obtained by GGAs using Perdew-Zunger parameterization [21] of the local density approximation (LDA) for exchange and correlation potential. As a result of this procedure, our theoretical band gap achieves a value very close to the experimental data.

In order to assess the possibility of doping a monolayer MoS_2_ with particular impurities or creating vacancies, we calculate the corresponding formation energy in the presence of the defect. For a substitutional dopant *D* in a MoS_2_ monolayer, the formation energy [22] *E*^*f*^(*D*) is defined as:
1

where  is the total energy of the system with a substitutional or absorbed dopant *D*, and *E*_tot_(*MoS*_2_) is the total energy of the system of pristine monolayer MoS_2_, while *μ*_host_ and *μ*_*D*_ denote the chemical potentials of host atoms (Mo, S) and dopant atom *D,* respectively*.* The reference levels of calculated chemical potentials are bulk (D_2_) for Mo (S) dopants or defects. When the dopant is an adsorbate, there is no *μ*_host_ term in (1). For Mo(S) vacancy *V*_Mo(*S*)_ in MoS_2_, the formation energy is instead obtained by the below expression:
2

where  is the total energy of the defective MoS_2_ with Mo (S) vacancy.

## Results and discussion

**Figure 2:**
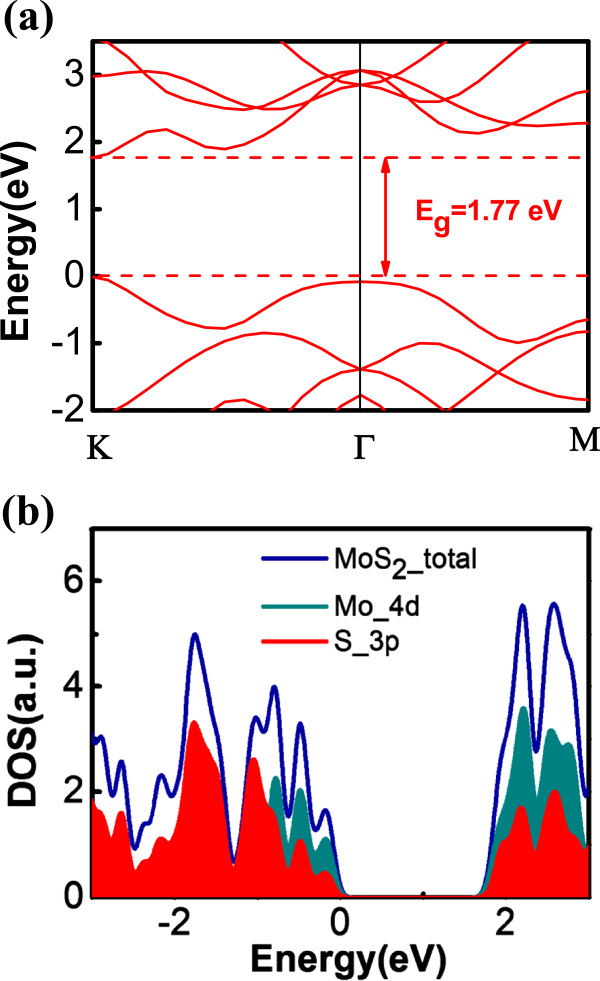
**Band structure and DOS plot of monolayer MoS**_**2**_ **. (a)** Band structure of monolayer MoS_2_ with the CBM and VBM both at the K point with a direct gap = 1.77 eV. **(b)** DOS of pristine MoS_2_ single layer with DOS projected on Mo 4d orbital and S 3p orbital.

The computed band structure of MoS_2_ by using LDA is shown in Figure 2a. One notices that the valence band maximum (VBM) and conduction band minimum (CBM) are both located at the K point of the Brillouin zone, which indicates a direct bandgap. The valence band top originates mainly from the 4d states of Mo atom, while the conduction band bottom consists of both 4d states of Mo and 3p states of S atoms, as shown in the partial DOS plot in Figure 2b, in agreement with the previous work [23].

**Figure 3:**
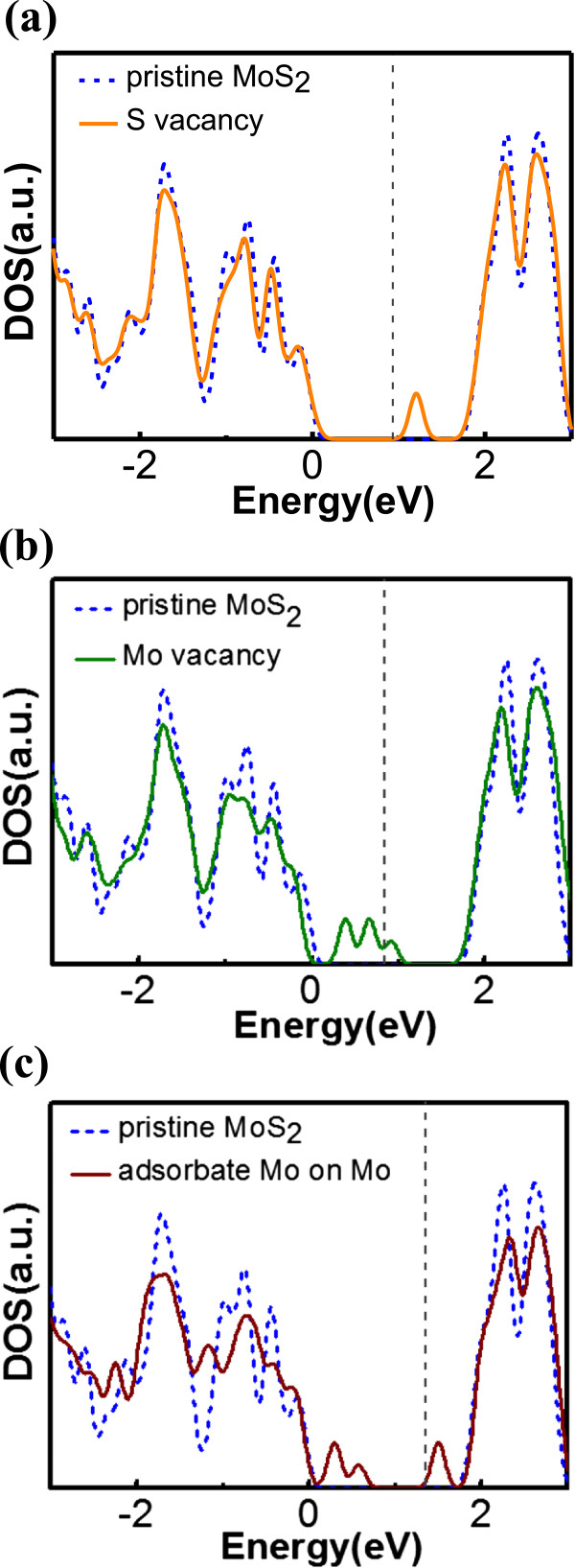
**DOS of MoS**_**2**_ **with (a) S vacancy (b) Mo vacancy, and (c) adsorbate Mo on Mo (solid line).** Pristine MoS_2_ (dashed blue line). *E* = 0 is the VBM. The vertical grey dashed line stands for Fermi level.

Before studying the doping effect of substitutional impurities on monolayer MoS_2_, we first simulate the electronic properties of vacancies in MoS_2_ by removing a Mo or an S atom from a pristine MoS_2_ monolayer to identify the corresponding gap states and assess the existence of a resulting magnetic moment. Formation energies are calculated for the purpose of estimating the tendency of vacancy creation and listed in Table 1 with theoretical magnetic moments. The DOSs for two kinds of vacancies are also displayed in Figure 3. As revealed from our formation energy calculation, creating an S vacancy (*E*_form_ ≈ 3.36 eV) is more energetically favorable than creating a Mo vacancy (*E*_form_ ≈ 7.36 eV), which is in good agreement with the experimental findings [24]. Furthermore, the DOS plots in Figure 3a show the gap states generated by a single S vacancy are close to the conduction band, while the states originating from single Mo vacancy (Figure 3b) are in even closer proximity of the VBM with three peaks of gap states arising from the mixture of the neighboring S p-orbitals. This suggests the possibility of p-type doping if Mo vacancy can be created in an efficient way. However, there is no magnetism induced in monolayer MoS_2_ by Mo or S vacancy as found in our simulation, showing no reason of using intrinsically defective MoS_2_ for spintronics applications, unlike the case of graphene and h-BN [25, 26]. In Figure 3c, we show the DOS for a Mo atom adsorbed on Mo site in MoS_2_ cell. It is seen that the incorporation of the adsorbate Mo creates defect states near the both band edges, VBM, and CBM, with a formation energy of approximately 1.1 eV higher than for an S vacancy, but not as high as for a Mo vacancy. Therefore, in addition to S vacancy, the Mo adsorbate can be a possible source of tail states as observed in the experiment [27].

**Table 1:** **Calculated formation energy (** ***E***_**form**_ **) for Mo-rich condition and the magnetic moment (m) of various impurities and defects in MoS**_**2**_ **monolayers**

Impurity/defect	m(μ _B_/impurity)	***E***_form_(eV)
Mo vacancy	0	7.36
S vacancy	0	3.36
Mo adatom on Mo	0	4.50
N	1.00	2.78
P	1.00	1.95
As	1.00	2.04
Mn	1.00	1.78
Fe	2.00	2.43
Co	3.00	3.72
V	1.00	0.16
Nb	0.52	−0.28
Ta	1.00	−0.35
C	0.00	0.14
Si	0.00	1.56
Ge	0.00	2.25
B	1.00	0.22
Al	1.00	2.04
Ga	1.00	1.97

**Figure 4:**
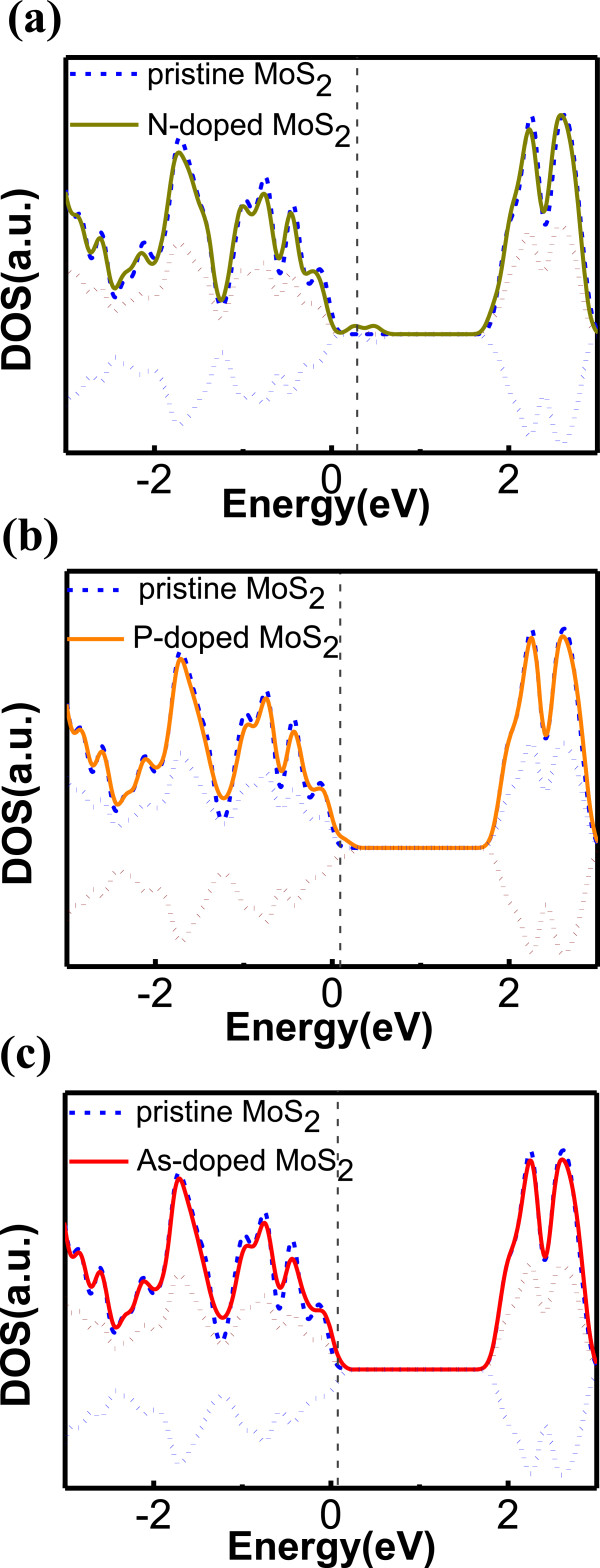
**DOS of MoS**_**2**_ **doped with (a) N (b) P, and (c) As (solid line).** Positive (negative) dotted lines: up-spin (down-spin) states. Pristine MoS_2_ (dashed blue line). *E* = 0 is the VBM. The vertical grey dashed line stands for Fermi level.

Next, we turn our analysis to the properties of dopants substituted at the S site, beginning with the replacement of an S atom with VA group elements, such as N, P, and As. As shown in Figure 4a for N-doping, we can see that the impurity states are near the valence band edge with two spin states separated by nearly 0.2 eV and a magnetic moment of 1 μ_B_. For P-doped MoS_2_ (Figure 4b), the similarity with N impurity can be observed as the system is again magnetic, and the gap states move even closer to the valence band. Going down the periodic table further, the DOS of MoS_2_ with As impurity shows acceptor states in the gap almost merging with the valence band states of pristine MoS_2_ (Figure 4c), it therefore indicates that As can be a suitable p-dopant in MoS_2_. The formation energies for all three elements, all around 2 eV, are rather large, i.e., those elements are unlikely to be stable dopants at thermal equilibrium, therefore the technique of creating S vacancies may be a better option [24]. Here, we observe the impurity states shift to the valence band as atomic number increases, which is consistent with previous theoretical work by Dolui et al. [28]. Also the formation energies in our two works are in good agreement (approximately 5%). However, we also find MoS_2_ systems doped with an As atom exhibits a magnetic moment of 1 μ_B_, as with N and P dopants, which was not reported previously. This is possibly due to the slightly larger lattice constant of our MoS_2_ model.

**Figure 5:**
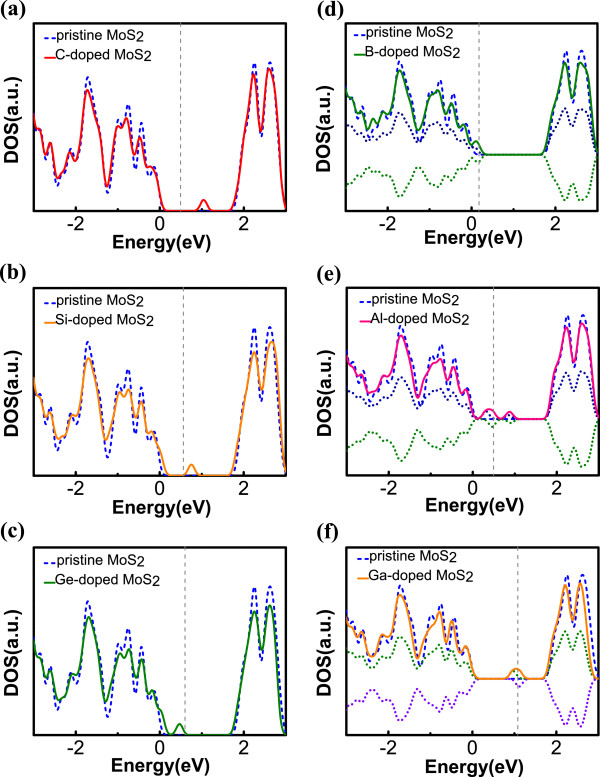
**DOS of MoS**_**2**_ **doped with (a) C (b) Si (c) Ge (d) B (e) Al, and (f) Ga (solid line).** Positive (negative) dotted lines: up-spin (down-spin) states. Pristine MoS_2_ (Dashed blue line). *E* = 0 is the VBM. The vertical grey dashed line stands for Fermi level.

To further assess the possibility of p-type doping by the replacement of S with other group A elements, we choose to study groups III and IV elements, such as C, Si, Ge, B, Al, and Ga that are commonly used in today’s industry. When a C, Si, or Ge atom replaces an S atom, there is a deficit of two valence electrons/atom, but the two remaining electrons have opposite spins, making the system nonmagnetic (Table 1). When substituting C for S, our calculation shows that a mid-gap state appears well above the Fermi level, acting more as a recombination center than as a doping state. For Si dopant, two defect states arise within the bandgap, one at valence band edge and the other close to mid-gap at 0.75 eV from the valence band (Figure 5a). However, the band edge state is already occupied, leaving only the mid-gap state acting as p-dopants or as electron traps, thereby hardly contributing any doping effect as well. In the case of Ge, similar to Si dopants, there are two defect states, with the mid-gap state shifting toward the valence band by approximately 0.3 eV (Figure 5b). Yet at room temperature, it is still quite unlikely to thermally generate a significant amount of free holes through such a state located so far from the VBM.

For group III elements, such as B, Al, and Ga atoms, all of them have three valence electrons less than S atoms. Our calculations show that each of these doped systems has only one remaining unpaired electron, resulting in a magnetic moment of 1 μ_B_ (Table 1). Figure 5c,d,e show the DOSs of B, Al, and Ga doped MoS_2_, with all these systems forming two gap states with opposite spins. The two states formed by the boron substitution in MoS_2_ are relatively close to the VBM with the partially unoccupied down-spin state separated from the VBM by less than 0.1 eV and exhibiting a spin splitting as small as 0.07 eV. Thus our result shows that the separation between these impurity states and the VBM is less than 50% of the data reported by Qu Yue et al. [29] (approximately 0.3 eV), which is due to the fact that we use a larger computational cell, thereby resulting in a more accurate binding energy. With an increasing atomic number, group III dopants are characterized by a different trend than group V dopants. The B doping induces impurity states located closest to the VBM than any other groups III and IV dopants. Although these states are slightly higher than that of As, the much lower formation energy of B doping still makes it a highly promising choice for achieving p-type doping in MoS_2_. When considering Al and Ga dopants, the defect states shift further away from the VBM with large energy differences, i.e., 0.25 eV for Al and 0.9 eV for Ga, and the formation energy also increases from 0.22 eV to around 2 eV (Table 1). To summarize our calculations on the doping at the S site, we find the two smallest atoms (B, C) in our study require the lowest two formation energies. This is because if the size difference between the dopant atoms and Mo are larger, the dopants will be bonded closer and stronger to Mo and the more negative binding energies subsequently cause the lower formation energies. On the other hand, the hybridization between p-orbital of dopant atoms and d-orbital of Mo leads to the impurity states, like the mechanism of Mo-S bonding. Therefore, to introduce gap states near the edge of valence band, the dopants must bring about a valence band structure very similar to what S does. In our work, P, As, and B do the best jobs and prove our theory by showing the closest gap states to VBM.

**Figure 6:**
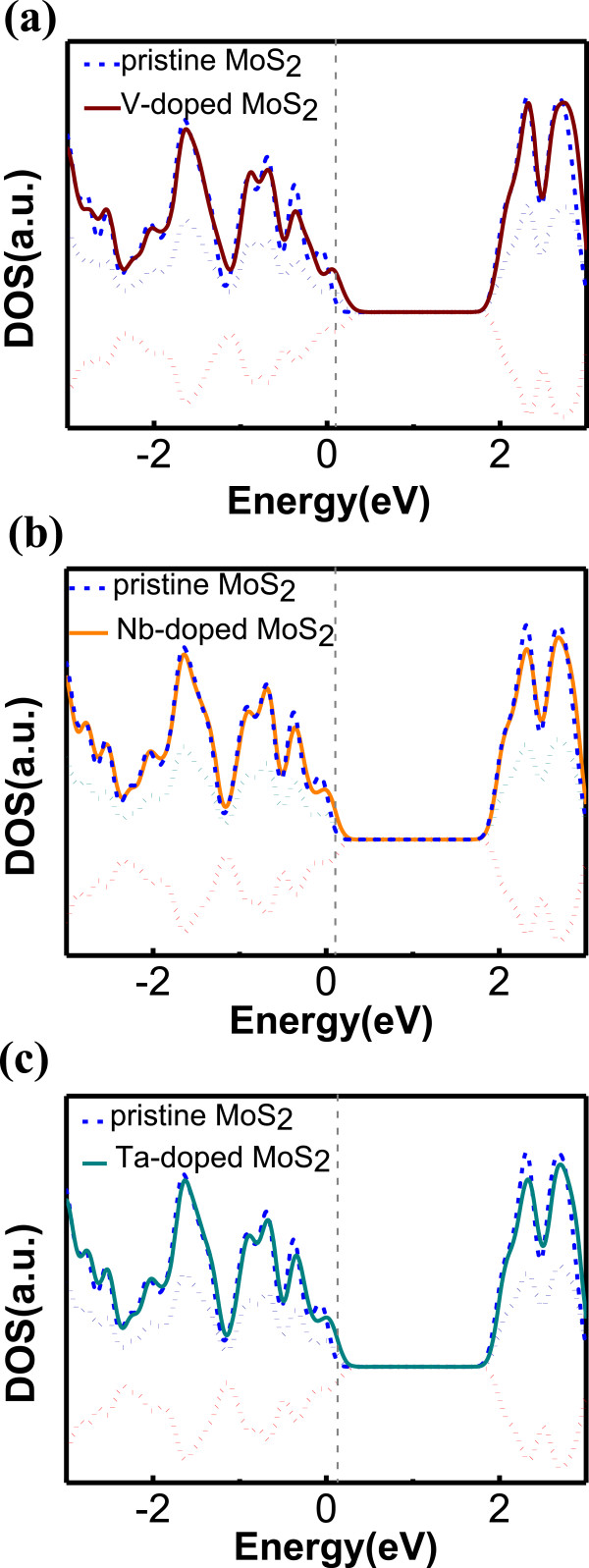
**DOS of MoS**_**2**_ **doped with (a) V (b) Nb, and (c) Ta (solid line).** Positive (negative) dotted lines: up-spin (down-spin) states. Pristine MoS_2_ (dashed blue line). *E* = 0 is the VBM. The vertical grey dashed line stands for Fermi level.

Next, we study the substitution of a Mo atom by different transition metal (TM) atoms. To start with, we consider the VB group elements that have one valence electron less than Mo and a magnetic moment of 1 μ_B_ (0.52 μ_B_ for Nb), which suggests the possibility for p-type doping. The DOS plots for MoS_2_ doped with three different elements V, Nb, and Ta are shown in Figure 6a,b,c. The impurity states originating from the three dopants are sitting at less than 0.1 eV away from the VBM, all of them characterized by a small spin-splitting. The formation energies for this type of dopants are also very low, decreasing with increasing atomic number. The most interesting feature is that under Mo-rich condition, i.e., when the Mo chemical potential is equal to its bulk Mo value, the formation energies become negative with Nb and Ta down in the VB column. As displayed in Table 1, Ta doping shows the lowest formation energy ever in our study, i.e., *E*_form_ ≈ −0.35 eV, whereas the formation energy of Nb is slightly higher, i.e., *E*_form_ ≈ −0.28 eV but is lower than the previous calculation [28], thanks to our larger cell size. Our model also shows that these values can be even lower in the S-rich limit [30]. Contrary to the case of S replacement, now the formation energies gets lower as atom size gets bigger, which results from the more significant size difference between these dopants and S atom. And all three elements induce very similar valence band structures to that of pristine MoS_2_, especially Ta. Not only are our findings in agreement with the previous work of Dolui et al. [28] but they also predict the VB group elements are optimal candidates for achieving p-type doping in MoS_2_.

**Figure 7:**
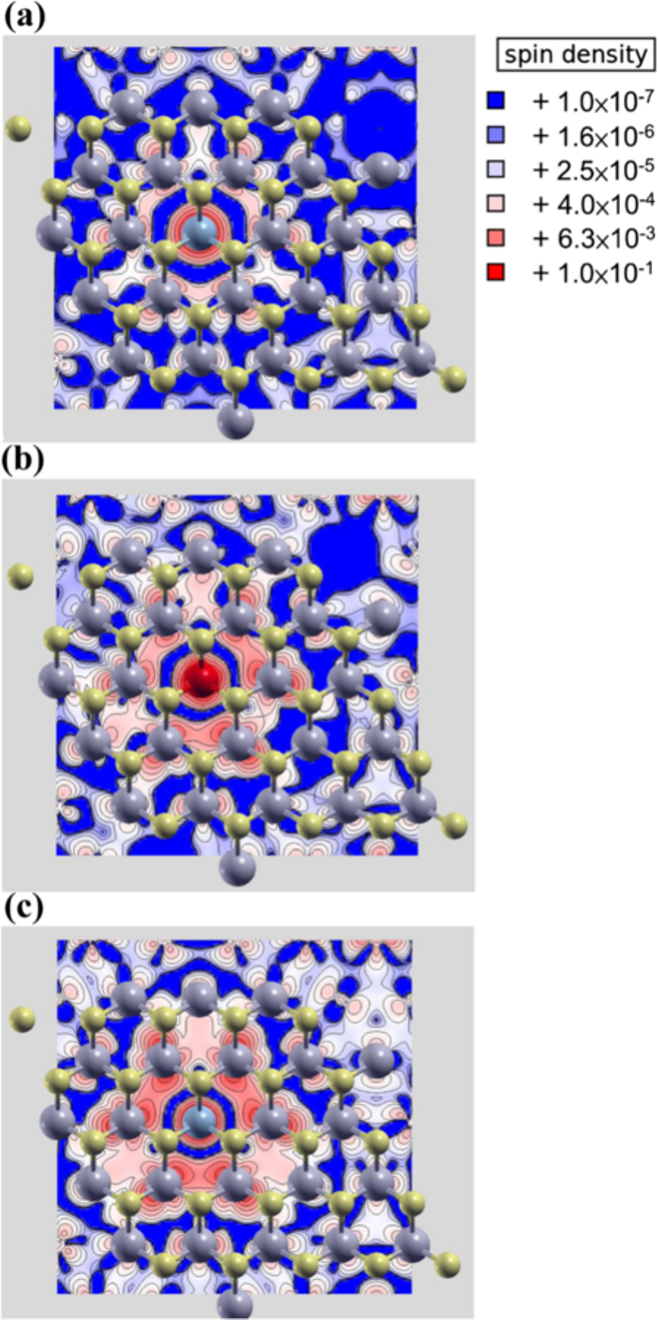
**Spin density of (a) Mn (b) Fe, and (c) Co-doped MoS**_**2**_ **.**

**Figure 8:**
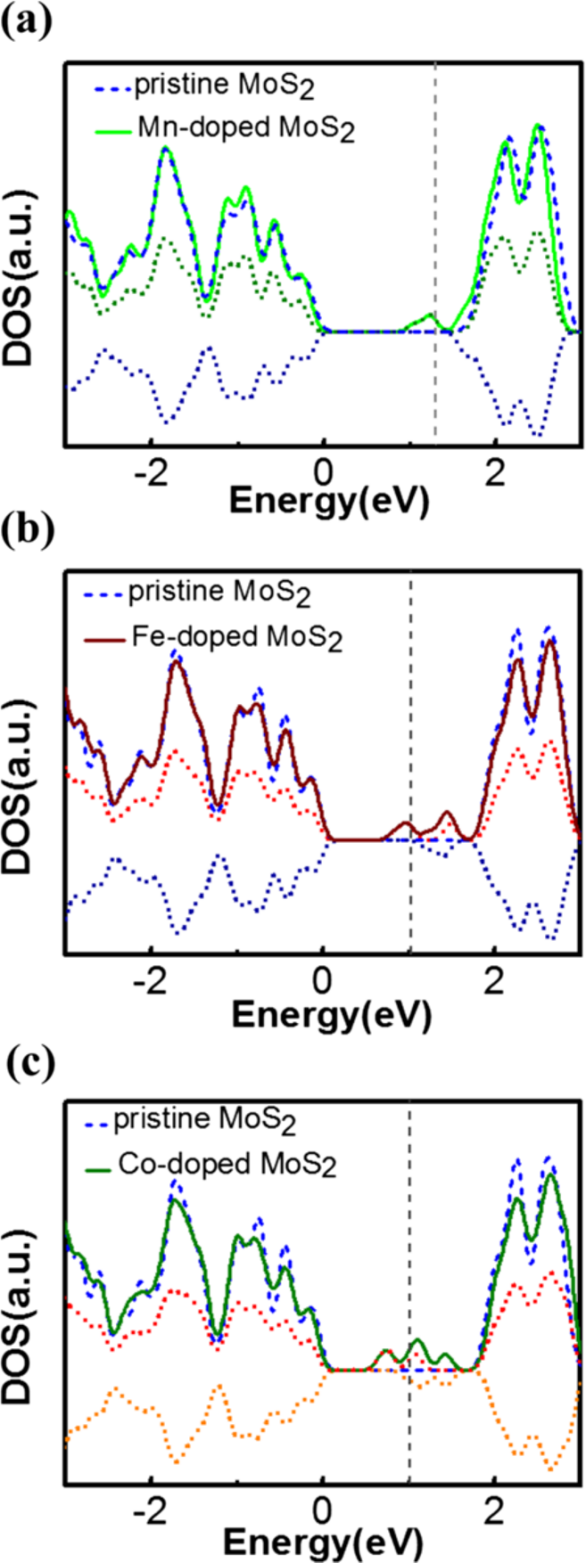
**DOS of MoS**_**2**_ **doped with (a) Mn (b) Fe, and (c) Co (solid line).** Positive (negative) dotted lines: up-spin (down-spin) states. Pristine MoS_2_ (dashed blue line). *E* = 0 is the VBM. The vertical grey dashed line stands for Fermi level.

Lastly, we consider magnetic TM elements, such as Mn, Fe, and Co, as representative of the VIB, VIIB, and VIIIB group elements that have, respectively, one, two, and three electrons in excess to Mo atoms. In Figure 7a, the spin density plot shows the most localized distribution around the Mn atom with a total magnetic moment of 1 μ_B_. For Fe and Co (Figure 7b,c), although the total magnetic moment is stronger, i.e., 2 and 3 μ_B_, respectively, the distribution of spin density becomes less localized and broader with the spins spreading to the neighboring atoms in both cases. Despite the broadened spin distribution, all three elements still have higher spin density than that of V, Nb, and Ta. The DOSs of these doped systems are shown in Figure 8a,b,c, where we find another noticeable effect of the Mn atom, as its impurity level is the nearest to the conduction band, whereas Fe and Co dopants induce impurity levels much farther away from the CBM. Because of the use of a 5 × 5 cell, the impurity levels of all three elements are closer to the CBM than the recent reports using 4 × 4 cell [17, 18]. Moreover, for Mn, Fe, and Co dopant atoms, the separation between Fermi level and the conduction band is 0.48, 0.77, and 0.78 eV, respectively, which is also narrower than the previous calculation, making Mn dopant more attractive for n-type doping. The Mn doping is also the most energetically favorable (*E*_form_ ≈ 1.78 eV) among these magnetic impurities, as the formation energy increases with the atomic number, which is due to the smaller size difference between dopants and S, consistent with our prediction. As a result, Mn shows the highest potential for spintronics applications among those possible candidates as well as the capability of obtaining n-type doping in MoS_2_.

## Conclusions

We used density functional theory over a 5 × 5 atomic cell to investigate systematically the electronic properties of various impurities, vacancies, adatoms, and magnetic impurities in monolayer MoS_2_, including groups III and IV dopants, as well as magnetic TM atoms such as Mn, Fe, Co, V, Nb, and Ta. Without being computationally prohibitive, the use of 5 × 5 atomic cells provide a more accurate description of the band structure than the single MoS_2_ cells by relaxing the boundary conditions on the electronic wave functions. Specifically, by investigating the density of states and formation energy of single atom vacancies as well as adatoms in MoS_2_, we found that the Mo adatom can be a possible source of tail states as reported in experiment. In addition, the use of a 5 × 5 cell indicates that the B dopants in MoS_2_ induce impurity states that is very close to the VBM, with a low formation energy around 0.2 eV, which is the second lowest among the column III ~ V dopants, and is therefore a highly promising candidate for achieving p-type doping in monolayer MoS_2_. Our results also indicate that VB group impurity elements such as V, Nb, and Ta have very low formation energies for their substitution of a Mo atom in MoS_2_, achieving the most negative value for Ta, which has not been reported before. All these impurity states sit at less than 0.1 eV from the VBM, making them optimal candidates for p-type dopants in MoS_2_. Lastly, when considering Mn, Fe, and Co, with 1, 2 to 3 magnetic moment per atom, respectively, our 5 × 5 atomic cell model provides spin distribution of the three elements that becomes less localized as atomic number increases. Among them, Mn has the lowest formation energy, which makes it a potential candidate for spintronics applications of MoS_2_.
